# Anti‐tumor effects of miR‐34a by regulating immune cells in the tumor microenvironment

**DOI:** 10.1002/cam4.5826

**Published:** 2023-03-23

**Authors:** Man Yin, Zhiqiang Zhang, Yunfei Wang

**Affiliations:** ^1^ Department of Clinical Medicine Jining Medical University Jining Shandong 272000 China; ^2^ Department of Gynecology Affiliated Hospital of Jining Medical University Jining Shandong 272029 China

**Keywords:** antitumor, immunotherapy, miR‐34a, tumor immune microenvironment

## Abstract

Malignant tumors pose a serious threat to human health. The development of malignant tumors is characterized by uncontrolled cell division and immune evasion. The micro‐ribonucleic acid‐34a (miR‐34a) is a small noncoding single‐stranded ribonucleic acid that is ubiquitously present in normal human tissues. However, it has been confirmed to be dysregulated in a variety of tumor cells. Numerous research have revealed the importance of miR‐34a in the treatment of various malignancies. MiR‐34a deletion can hasten the growth of tumors whereas miR‐34a overexpression suppresses the proliferation, invasion, and migration of cancer cells. Moreover, more recent studies have highlighted its role in immunity and investigated its applicability to particular tumors. Through various immune cells, factors, and other mechanisms, miR‐34a can inhibit tumor carcinogenesis. In view of the important role of miR‐34a in tumors, this research reviewed the aspects of miR‐34a regulation of tumor immune microenvironment to exert anti‐tumor effects in order to clarify the potential immunotherapy value of miR‐34a in tumors.

## Graphical abstract

The onset and progression of cancer are significantly influenced by the tumor microenvironment. miR‐34a has been confirmed to be dysregulated in a variety of tumor cells. This manuscript summarizes the aspects of how miR‐34a can play an anti‐tumor role by regulating the tumor immune microenvironment, thereby clarifying the potential immunotherapeutic value of miR‐34a for tumors.

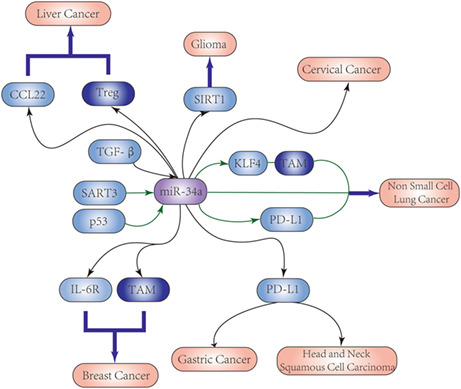

## 1 INTRODUCTION

Immunotherapy is currently being used more and more often to treat malignant tumors. Immunotherapy strategies such as tumor vaccines and immune checkpoint inhibitors have achieved substantial therapeutic effects and achievements. Numerous studies are trying to identify genes involved in regulating the tumor microenvironment that could be safely targeted by these drugs. Small noncoding single‐stranded ribonucleic acid (RNA), known as microRNA (miRNA), are found in large quantities in eukaryotes.^1^ These molecules have an important role in the control of vital cellular processes such as the cell cycle, differentiation, and death.^2^, ^3^ Numerous studies have demonstrated that the majority of malignant tumors have dysregulated miRNA expression, which can lead to the initiation and growth of malignancies.^4^, ^5^, ^6^ The micro‐ribonucleic acid‐34(miR‐34) family is one of the three major miRNA families. The miR‐34 family is made up of three molecules: miR‐34a, miR‐34b, and miR‐34c.^7^ Numerous malignant tumors lack its expression, including lung cancer, pancreatic cancer, and prostate cancer.^8^ Apoptosis and senescence are two processes that are inhibited by an increase in miR‐34a, which prevents carcinogenesis and cancer growth when it is inhibited. Relevant studies have indicated that miR‐34a may be a prospective candidate gene for cancer immunotherapy. This paper provides a review of studies related to how miR‐34a regulates the tumor immune microenvironment to influence cancer progression in various cancers.

## 2 THE EFFECT OF MIR‐34A ON THE TUMOR IMMUNE MICROENVIRONMENT

### 2.1 Tumor immune microenvironment

**FIGURE 1:**
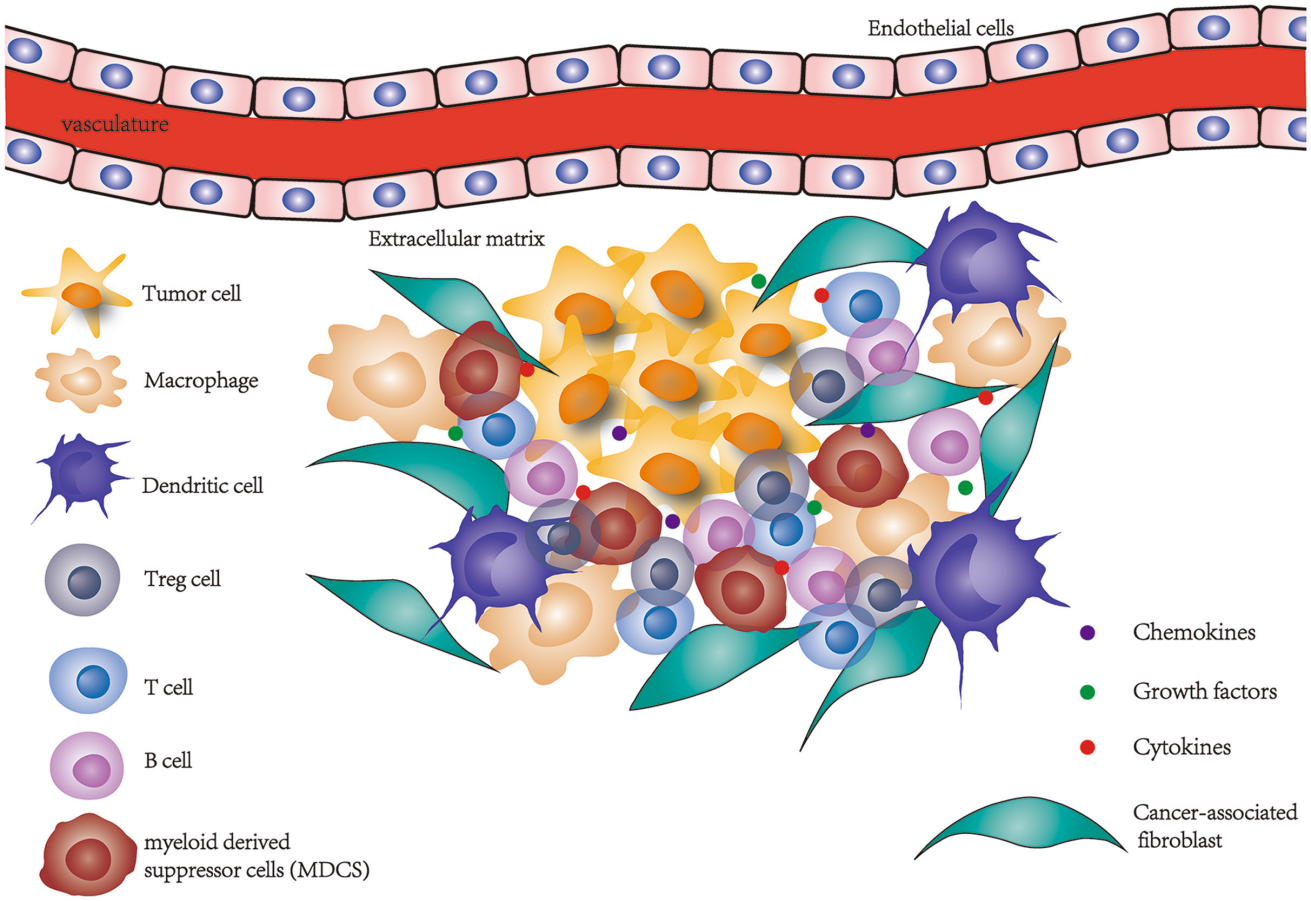
Composition of tumor microenvironment.

The tumor microenvironment surrounds tumor tissues and capillaries, and consists of tumor cells, fibroblasts, immune and inflammatory cells, glial cells, various cytokines, and chemokines (Figure 1). Due to its significance in the initiation, growth, progression, and prognosis of cancer, the tumor immune microenvironment has recently emerged as one of the most prestigious disciplines in cancer research. Some studies have explored the function of the immune microenvironment in regulating immunotherapy and the prognosis of patients.^9^ miR‐34a has an important role in regulating the production and activation of various immune cells, including the tumor‐infiltrating lymphocytes (TILs), CD8^+^ cytotoxic T lymphocytes, regulatory T cells (Tregs), tumor‐associated macrophages (TAMs), and the myeloid‐derived suppressor cells (MDSCs).

### 2.2 TAMs

Macrophages develop from monocytes. Monocytes circulate in the bloodstream and can migrate out of the bloodstream into tissues and differentiate into macrophages under the influence of the local microenvironment. TAMs are the fundamental constituents of the tumor microenvironment.^10^ TAMs can penetrate the tumor immune microenvironment and promote immunosuppression, angiogenesis, chemical tolerance, tumor progression, and metastasis. There are different subpopulations of macrophages, including CD169(+) macrophages and TCR (+) macrophages, in addition to TAMs that can be polarized into M1 or M2 macrophages.^11^ M1 macrophages are typically activated by pro‐inflammatory cytokines and are responsible for developing an inflammatory response and the destruction of harmful bacteria and tumor cells. Conversely, M2 macrophages are activated by anti‐inflammatory cytokines and tissue repair factors, and can suppress the inflammatory response, induce tissue repair, and promote cancer development. TAMs are not present in normal conditions and their appearance is associated with specific pathological settings with M1 and M2 polarization. Macrophage polarization and penetration into the tumor microenvironment are key determinants of tumor progression.

There has been research into how miR‐34a affects macrophage differentiation. For example, miR‐34a‐expressing MDA‐MB‐231 breast cancer cells can more potently cause THP‐1 monocytes to polarize into M1 macrophages. Anti‐miR‐34a transfection caused M2 macrophages to form in cancer cells while inhibiting M1 macrophage development.^12^ Similar effects were observed for head and neck cancer,^13^ uterine leiomyosarcoma,^14^ and lung cancer.^15^ The role of TAM polarization in tumor formation and progression is an active area of research, as it is believed to play a critical role in modulating the tumor microenvironment.

### 2.3 MDSCs

Numerous immunosuppressive cytokines, including the transforming growth factor beta (TGF‐β), interleukin‐10 (IL‐10), vascular endothelial growth factor, and prostaglandin E2, are secreted by tumor cells. The presence of these cytokines in the tumor immune microenvironment can stimulate bone marrow precursor cells to differentiate into dendritic cells (DCs), macrophages, granulocytes, and mast cells. Although MDSCs and DCs share the same bone marrow precursor cells, they play very different roles in the immune response. While DCs are critical for the initiation and regulation of the tumor immune responses, MDSCs have an immunosuppressive function and can promote tumor growth and immune evasion.

MDSCs were first identified in tumor tissues and lymph nodes of tumor‐bearing mice^16^ and represent a mixed population of immature bone marrow cells, including DC precursors. Relevant studies^17^ have shown that the ability of bone marrow precursors to enter MDSCs is influenced by variations in miR‐34a levels in liver cancer and colon cancer cells. As a result, miR‐34a upregulation decreases the capacity of the tumor supernatant to stimulate bone marrow precursors to enter MDSCs. Conversely, the downregulation of miR‐34a can increase the ability of tumor supernatant to induce bone marrow precursors to enter MDSCs. Additionally, TGF‐β and IL‐10 have been shown to induce the entrance of bone marrow precursor cells into MDSCs. These cytokines can activate signaling pathways that promote the differentiation and expansion of MDSCs, contributing to the immunosuppressive environment of the tumor microenvironment. Targeting these pathways and molecules involved in the differentiation and function of MDSCs could provide a potential strategy for the development of cancer immunotherapy.

### 2.4 TILs

TILs are immune cells in the microenvironment of tumor tissue, indicating the presence of antitumor immune responses.^18^ CD4^+^ and CD8^+^ T cells are the two main types of TILs. The cytotoxic CD8^+^ T lymphocytes are activated by proteins in tumor cells. Upon activation, the CD8^+^ cells release cytotoxic molecules that induce cell death. Tregs are responsible for suppressing the activity of CD8^+^ cells through the deactivation of antigen‐presenting cells via the downregulating of costimulatory proteins.^19^ Therefore the subpopulation of TILs is often used clinically to predict treatment outcomes. Researchers have found that^20^ miR‐34a may regulate the activity of CD4^+^ T cells in triple‐negative breast cancer (TNBC) by controlling the expression of specific genes that influence T‐cell infiltration into tumors.

### 2.5 Tregs

FoxP3‐expressing Tregs are often the major immune system inhibitors in the tumor microenvironment and actively uphold immunological homeostasis and self‐tolerance by suppressing a variety of immune responses. Treg cells often accumulate in tumor masses and ascites.^21^ Recent research has shown that several chemokines can attract Treg cells to control the immune responses during cancer.^22^ MiR‐34a exerts its cellular nonautonomous tumor suppressor effect by regulating the expression of the chemokine CCL22 and the recruitment of Treg cells. The migratory activity of Treg cell is increased when miR‐34a is inhibited, demonstrating that increased Treg recruitment is linked to decreased miR‐34a expression.

## 3 ROLE OF MIR‐34A IN THE REGULATION OF ANTI‐TUMOR IMMUNITY AND THE TUMOR IMMUNE MICROENVIRONMENT

**FIGURE 2:**
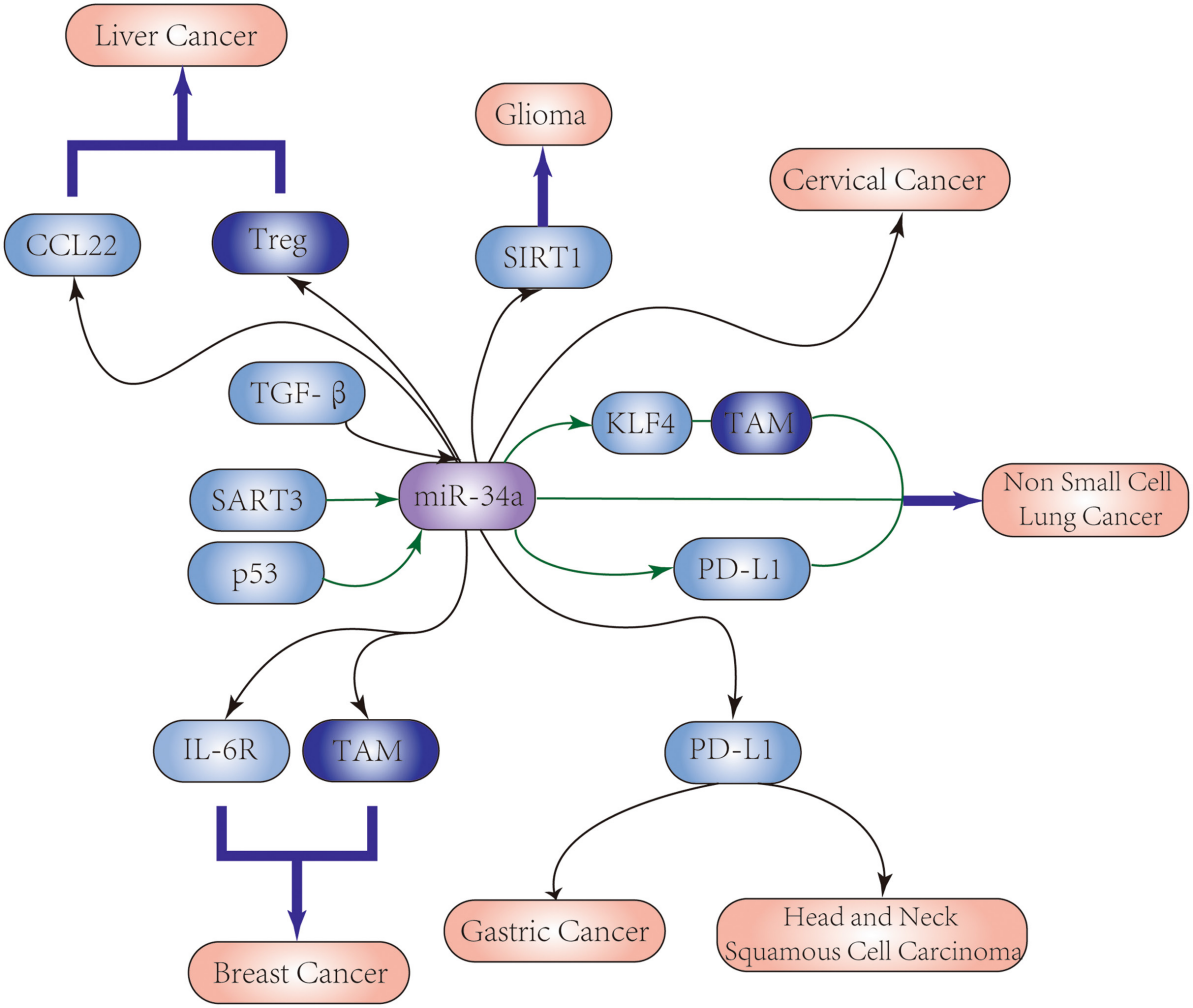
Targeted pathways of miR‐34a in the immune microenvironment of many cancers.

MiR‐34a plays a key role in regulating the immunological microenvironment of many malignancies, including breast, gastric, lung, glioma, liver, cervix, and head and neck cancer (Figure 2; Table 1).

**TABLE 1:** List of miR‐34a targets in the tumor microenvironment of various cancers.

Cancer type	Immuno	Involved molecule	Involved target	Function	Reference
Breast cancer	TAM/M2	IL‐6R	MCT‐1	Reprograms EMT and macrophages as well as inhibits tumor progression	^12^
	CD4^+^ T cell	CAPN6	/	Regulates cellular activities associated with CD4+ T cell infiltration	^20^
Gastric cancer	T, B, and NK cells	PD‐L1	/	Suppresses the proliferation as well as the migration and invasion of tumor	^23^
Lung cancer	TAM	KLF4	/	Modulation of macrophage polarization reverses the processes of tumorigenesis	^15^
	macrophages and Treg cells	PD‐L1	p53	Regulates tumor immune evasion	^24^
Glioma	MSCs	SIRT1	p53, Cdkn1a, and Cdkn2c	Induces glioma cell senescence	^25^
Liver cancer	Treg cells	CCL22	/	Promotes tumor cells to escape from immune surveillance	^26^
Head and neck squamous cell carcinoma	pro‐B cells, CD8 naïve T cells, Th1 cells	PD‐L1	MET	Suppresses oncogenic MET and restores tumor immunity	^13^

### 3.1 MiR‐34a and breast cancer

Breast cancer is one of the most prevalent tumors and the second most common cause of cancer‐related deaths in women worldwide, accounting for around 25% of all female cancer cases. Despite the advancements in detecting and treating breast cancer, the clinical results remain dismal, with low 5‐year survival rates for women with metastatic breast cancer. The miR‐34 family includes tumor suppressor proteins that promote apoptosis, restrict cell migration and proliferation, and regulate the p53 signaling pathway.^27^, ^28^ There are few therapeutic options available for TNBC, a breast cancer with a dismal prognosis that lacks the estrogen receptor, progesterone receptor, and human epidermal growth factor receptor 2. The majority of TNBC cases also include p53 mutations.^29^, ^30^ Although miR‐34a was found to be highly expressed in healthy human mammary epithelial cells, its expression was inhibited in some TNBC cells known as MDA‐MB‐231 cells.^12^ Multiple Copies in T‐cell Malignancy 1 (MCT‐1) can destabilize p53,^31^ and Yueh‐Shan Weng et al. demonstrated that miR‐34a could inhibit IL‐6R expression in the MCT‐1 pathway. In addition, the researchers also co‐cultured TNBC cells transfected with miR‐34a with monocytes THP‐1, they found that MDA‐MB‐231 cells overexpressed with miR‐34a made monocytes more likely to polarize into M1‐type macrophages. These findings imply that miR‐34a is crucial for controlling the immunological microenvironment in TNBC. Not only that, some investigators performed gene expression analysis of T‐cell infiltration in breast cancer, and the results showed^20^ that miR‐34a may be associated with CD4^+^ T‐cell infiltration, which may provide new target ideas for immunotherapy of breast cancer. In addition, Chen et al.^32^ showed that the stem gene LIN28B promotes MYC expression and suppresses miR‐34a levels, thereby promoting glycolysis and affecting the prognosis of breast cancer patients. Additionally, evidence for it exists that this hypothesis was further supported by Xiao et al.,^33^ who also showed that miR‐34a overexpression might directly inhibit the ability of breast cancers to grow and metastasize via reducing the production of the lactate dehydrogenase enzyme responsible for glycolysis in cancer cells.

### 3.2 MiR‐34a and gastric cancer

Gastric cancer has become the most challenging malignant cancer; it has the fifth highest morbidity and third highest mortality worldwide.^34^ The development of gastric cancer has been linked with *Helicobacter pylori* infection, genetic variation and environmental factors.^35^, ^36^ Currently, even though significant improvements have been made in the areas of surgery, chemotherapy, radiotherapy, and immunotherapy, the 5‐year survival rate is still poor due to the difficulty of early detection and early diagnosis of gastric cancer, which can easily metastasize to other organs.^37^ Therefore there is a need to identify novel biomarkers that can be used for the early detection, diagnosis, and therapeutic targets. Based on previous studies, miRNAs have been shown to influence gastric cancer tumorigenesis.^38^, ^39^, ^40^ Among them, miR‐34a^41^ antagonizes the development of gastric cancer. According to an experimental study by Yong et al.,^23^ it was found that the inhibition of the programmed death ligand 1 (PD‐L1), a crucial regulator of tumor immune evasion, can reduce the expression of miR‐34a and the proliferation, migration, and invasion of gastric cancer cells.^42^ These findings suggest that the overexpression of miR‐34a can inhibit the immune evasion of cancer cells.

### 3.3 MiR‐34a and lung cancer

Lung cancer is the most common cause of death worldwide, with the highest incidence and mortality rates. Smoking is widely acknowledged to be the primary cause of lung cancer.^43^ About 85% of instances of lung cancer among them are non‐small cell lung cancer (NSCLC). It has been found that *SART3* overexpression increases miR‐34a levels and thus affects the cell cycle of NSCLC. p53, in turn, regulates PD‐L1 through miR‐34a (targeting EGFR) and inhibits NSCLC tumor growth and metastasis.^24^, ^44^, ^45^ In addition, miR‐34a is seen as a potential target in the fight against lung cancer.^46^, ^47^ MiR‐34a targets the Krüppel‐like factor 4 to re‐educate M2‐type macrophages to M1‐type macrophages, as demonstrated by Shweta Arora et al.^15^ To further confirm the effect of miR‐34a on the immunological milieu of lung cancer, the researchers co‐cultured pulmonary cancerous cells implanted with miR‐34a with macrophages and observed a decrease in usual markers for M2 and an increase in characteristic markers for M1. These findings imply that miR‐34a can exert its tumor‐killing activity through influencing the polarization of TAMs in NSCLC. In addition, although smoking is the primary cause of lung cancer, roughly 25% of patients worldwide do not currently smoke, and patients with lung cancer who have never smoked have the seventh‐highest mortality rate of all cancer patients worldwide.^48^ According to Sui et al.,^49^ miR‐34a tends to be overexpressed in lung cancer patients who never smoked and may therefore contribute to the development of this disease.

### 3.4 miR‐34a and glioma

The most frequent primary malignant tumors of the adult central nervous system are gliomas, which are brain tumors caused by the malignant transformation of oligodendrocytes and astrocytes.^50^ Glioblastoma multiforme (GBM), the most prevalent kind, has a very bad prognosis.^51^ The typical course of treatment for gliomas involves the maximum amount of surgical resection, followed by radiotherapy and chemotherapy.^52^ Unfortunately, the complete surgical excision of malignant gliomas is difficult due to their diffuse nature. In addition, since GBM is insensitive to chemotherapy and radiotherapy,^53^, ^54^ tumor recurrence is inevitable in the vast majority of patients. In order to address this issue, there is a growing research trend toward the development of non‐viral biotechnologies that employ safe and effective gene and cell‐based treatments for gliomas. Mesenchymal stem cells (MSCs) can secrete a number of immunomodulatory compounds, such as cell chemokines. Relevant studies^25^ demonstrated that the transfer of miR‐34a from human bone marrow‐derived mesenchymal stem cells could be used to target and inhibit SIRT1 (an anti‐aging factor), thereby suppressing the proliferation, invasion, and migration of glioma. Moreover, it can also induce glioma cell senescence and increase DNA damage. Therefore the delivery of miR‐34a by MSCs may provide a new therapeutic approach for glioma. Rathod et al. demonstrated that compared with normal brain tissue, miR‐34a expression is lower in glioma stem cells and primary gliomas.^55^, ^56^ Similarly, analysis of the data by Yin et al.^57^ also showed that miR‐34a could inhibit the development of GBM cells in vitro *and* in vivo by controlling the expression of cell cycle‐related proteins and EGFR.

### 3.5 MiR‐34a and liver cancer

Hepatocellular carcinoma (HCC) is a serious health issue that affects people all over the world. Because HCC is diagnosed at the late stage, many HCC patients present with symptoms of intrahepatic metastasis or postoperative recurrence. The 5‐year survival rate is only approximately 33%. Numerous studies indicate that exposure to carcinogens or toxins, hereditary factors, or infection with the hepatitis B and C viruses (HBV, HCV) can contribute to the development of HCC. Relevant researches^26^ have discovered that the decline in miR‐34a expression is connected to HBV‐induced HCC throughout the development of liver cancer. The HBV infection changes the perihepatic milieu by increasing the level of TGF‐β, which block miR‐34a expression and trigger the production of CCL22. The Treg cells are then attracted toward the tumor, thus promoting tumor immune evasion and metastasis. These findings suggest that miR‐34a‐targeted therapy could potentially be used to treat HCC. However, further research is required to determine the exact mechanism of action of miR‐34a in HCC.

### 3.6 MiR‐34a and cervical cancer

Cervical cancer is the gynecological cancer that strikes women most frequently and fatally, ranking fourth.^1^ However, since cervical cancer is a widely heterogeneous disease, traditional cancer treatment methods, such as surgery, chemotherapy, and radiotherapy, are often ineffective due to the multifactorial characteristics of genetic heterogeneity and drug resistance. Recently, targeted cancer therapy has been considered to be a promising treatment method because of the high expression of surface receptors on cervical cancer cells. In HPV‐positive cells, downregulation of miR‐34 family expression has been reported, which may cause p53 instability and lead to the proliferation of tumor cells.^58^ Similarly, Wang et al.^59^ showed that miR‐34a expression was noticeably downregulated in both the serum and tumor tissue of cervical cancer patients. In contrast, miR‐34a overexpression may result in cell cycle arrest, decrease cell growth, and increase cell death.^60^ Widespread expression of PD‐L1, a miR‐34a target,^24^ in cervical cancer is a crucial mechanism by which the disease manipulates the immune system.^61^, ^62^ It has been shown^63^ that the combination of anti‐PD‐L1 antibody and miR‐34a can inhibit cervical cancer tumor progression, while the two can act synergistically. It has not yet been determined if miR‐34a and PD‐L1 are directly related to cervical cancer. However, due to their close connection with tumor development, miR‐34a has gradually become a key biomarker and target for anti‐cancer therapy in cervical cancer.^64^

### 3.7 MiR‐34a and head and neck squamous cell carcinoma (HNSCC)

MiR‐34a is a biomarker that can be utilized in HNSCC for both diagnosis and prognosis.^65^, ^66^ Studies have found a link between the downregulation of miR‐34a and the angiogenesis and cell proliferation in HNSCC.^67^ Additionally, Wu et al.^13^ found that miR‐34a levels were markedly decreased in the tumor tissue of HNSCC patients. On the other hand, patients with miR‐34a overexpression had improved immune function via the blocking of PD‐L1. MiR‐34a also controlled the cell proliferation, proto‐oncogene MET, and tumor suppressor function. Therefore miR‐34a may provide a direct target for the proto‐oncogene while retaining the anti‐tumor immune function.

## 4 CONCLUSION AND PERSPECTIVE

This paper reviewed the antitumor effect of miR‐34a through regulation of the immunosuppressive microenvironment. miR‐34a has potent antitumor effects. Numerous investigators have confirmed its effectiveness in many tumor cell lines through in vivo and in vitro experiments, enabling the creation of matching inhibitors or enhancers for use in clinical therapeutics. Due to the complexity and diversity of the internal environment for tumor cell survival, how miR‐34a can be used in immunotherapy has received extensive attention.

However, although studies on the combination of miR‐34a and immunotherapy continue to emerge, the translation of this research to clinical practice has been challenging. The high heterogeneity and complexity of the tumor microenvironment make it difficult to develop single effective drug targets. More research is required to understand the impact of miR‐34a on the regulation of the tumor immune microenvironment. One of the main challenges is the efficient delivery of miRNA‐based therapeutics to target tissues, as miRNAs have poor pharmacokinetics and require delivery systems that can protect them from degradation and facilitate their uptake into target cells. Finally, most in vivo studies have been conducted on tumor mouse models, which lack the complexity of human tumors. Treatments that work in mice may not be effective in humans. Although miR‐34a‐targeted therapies are showing great potential in preclinical studies, further research is required to validate its role in cancer biology and to optimize its delivery and efficacy as a therapeutic agent.

## AUTHOR CONTRIBUTIONS

**Man Yin:** Writing – original draft (equal). **Zhiqiang Zhang:** Writing – review and editing (supporting). **Yunfei Wang:** Conceptualization (equal); writing – review and editing (lead).

## FUNDING INFORMATION

This work was supported by the National Natural Science Foundation of China (81502255), Shandong Provincial Traditional Chinese Medicine Science and Technology Development Project (M‐2022242) and the Key R&D Program of Jining (2020YXNS026, 2022YXNS007).

## CONFLICT OF INTEREST STATEMENT

The authors have no relevant financial or non‐financial interests to disclose.

## ETHICS STATEMENT

As this work was a review of the literature, no ethics committee approval was required.

## Data Availability

Data sharing is not applicable to this article as no new data were created or analyzed in this study.

## References

[1] Kim VN , Han J , Siomi MC . Biogenesis of small RNAs in animals. Nat Rev Mol Cell Biol. 2009;10(2):126‐139. doi:10.1038/nrm2632 19165215

[2] Garofalo M , Croce CM . MicroRNAs: master regulators as potential therapeutics in cancer. Annu Rev Pharmacol Toxicol. 2011;51(1):25‐43. doi:10.1146/annurev-pharmtox-010510-100517 20809797

[3] Mendell Joshua T , Olson EN . MicroRNAs in stress signaling and human disease. Cell. 2012;148(6):1172‐1187. doi:10.1016/j.cell.2012.02.005 22424228 PMC3308137

[4] Garzon R , Marcucci G , Croce CM . Targeting microRNAs in cancer: rationale, strategies and challenges. Nat Rev Drug Discov. 2010;9(10):775‐789. doi:10.1038/nrd3179 20885409 PMC3904431

[5] Ling H , Fabbri M , Calin GA . MicroRNAs and other non‐coding RNAs as targets for anticancer drug development. Nat Rev Drug Discov. 2013;12(11):847‐865. doi:10.1038/nrd4140 24172333 PMC4548803

[6] Lionetti M , Musto P , Di Martino MT , et al. Biological and clinical relevance of miRNA expression signatures in primary plasma cell leukemia. Clin Cancer Res. 2013;19(12):3130‐3142. doi:10.1158/1078-0432.Ccr-12-2043 23613318

[7] Rokavec M , Li H , Jiang L , Hermeking H . The P53/Mir‐34 axis in development and disease. J Mol Cell Biol. 2014;6(3):214‐230. doi:10.1093/jmcb/mju003 24815299

[8] Bader AG . Mir‐34–a MicroRNA replacement therapy is headed to the clinic. Front Genet. 2012;3:120. doi:10.3389/fgene.2012.00120 22783274 PMC3387671

[9] Xu M , Li Y , Li W , et al. Immune and stroma related genes in breast cancer: a comprehensive analysis of tumor microenvironment based on the cancer genome atlas (Tcga) database. Front Med. 2020;7:64. doi:10.3389/fmed.2020.00064 PMC7066229 32195260

[10] Quail DF , Joyce JA . Microenvironmental regulation of tumor progression and metastasis. Nat Med. 2013;19(11):1423‐1437. doi:10.1038/nm.3394 24202395 PMC3954707

[11] Chávez‐Galán L , Olleros ML , Vesin D , Garcia I . Much more than M1 and M2 macrophages, there are also CD169(+) and TCR(+) macrophages. Front Immunol. 2015;6:263. doi:10.3389/fimmu.2015.00263 26074923 PMC4443739

[12] Weng YS , Tseng HY , Chen YA , et al. MCT‐1/MIR‐34a/IL‐6/IL‐6r signaling axis promotes EMT progression, cancer stemness and M2 macrophage polarization in triple‐negative breast cancer. Mol Cancer. 2019;18(1):42. doi:10.1186/s12943-019-0988-0 30885232 PMC6421700

[13] Wu X , Cheng YL , Matthen M , et al. Down‐regulation of the tumor suppressor MiR‐34a contributes to head and neck cancer by up‐regulating the met oncogene and modulating tumor immune evasion. J Exp Clin Cancer Res. 2021;40(1):70. doi:10.1186/s13046-021-01865-2 33596979 PMC7890893

[14] Zhang Z , Sun C , Li C , et al. Upregulated Melk leads to doxorubicin chemoresistance and M2 macrophage polarization via the MiR‐34a/JAK2/STAT3 pathway in uterine leiomyosarcoma. Front Oncol. 2020;10:453. doi:10.3389/fonc.2020.00453 32391256 PMC7188922

[15] Arora S , Singh P , Ahmad S , et al. Comprehensive integrative analysis reveals the association of KLF4 with macrophage infiltration and polarization in lung cancer microenvironment. Cell. 2021;10(8):2091. doi:10.3390/cells10082091 PMC8392240 34440860

[16] Gabrilovich DI , Nagaraj S . Myeloid‐derived suppressor cells as regulators of the immune system. Nat Rev Immunol. 2009;9(3):162‐174. doi:10.1038/nri2506 19197294 PMC2828349

[17] Wang X , Chang X , Zhuo G , Sun M , Yin K . Twist and MiR‐34a are involved in the generation of tumor‐educated myeloid‐derived suppressor cells. Int J Mol Sci. 2013;14(10):20459‐20477. doi:10.3390/ijms141020459 24129179 PMC3821625

[18] Miyan M , Schmidt‐Mende J , Kiessling R , Poschke I , de Boniface J . Differential tumor infiltration by T‐cells characterizes intrinsic molecular subtypes in breast cancer. J Transl Med. 2016;14(1):227. doi:10.1186/s12967-016-0983-9 27473163 PMC4966793

[19] Asano Y , Kashiwagi S , Goto W , et al. Tumour‐infiltrating CD8 to FOXP3 lymphocyte ratio in predicting treatment responses to Neoadjuvant chemotherapy of aggressive breast cancer. Br J Surg. 2016;103(7):845‐854. doi:10.1002/bjs.10127 26953091

[20] Wang Z , Yang X , Shen J , et al. Gene expression patterns associated with tumor‐infiltrating CD4+ and CD8+ T cells in invasive breast carcinomas. Hum Immunol. 2021;82(4):279‐287. doi:10.1016/j.humimm.2021.02.001 33612391

[21] Quezada SA , Peggs KS , Curran MA , Allison JP . CTLA4 blockade and GM‐CSF combination immunotherapy alters the Intratumor balance of effector and regulatory T cells. J Clin Invest. 2006;116(7):1935‐1945. doi:10.1172/jci27745 16778987 PMC1479425

[22] Curiel TJ , Coukos G , Zou L , et al. Specific recruitment of regulatory T cells in ovarian carcinoma fosters immune privilege and predicts reduced survival. Nat Med. 2004;10(9):942‐949. doi:10.1038/nm1093 15322536

[23] Yong H , Fu J , Gao G , Shi H , Zheng D , Zhou X . MiR‐34a suppresses the proliferation and invasion of gastric cancer by modulating Pdl1 in the immune microenvironment. Mol Cell Probes. 2020;53:101601. doi:10.1016/j.mcp.2020.101601 32445780

[24] Cortez MA , Ivan C , Valdecanas D , et al. PDL1 regulation by P53 via miR‐34. J Natl Cancer Inst. 2016;108(1):djv303. doi:10.1093/jnci/djv303 26577528 PMC4862407

[25] Li Q , Wang C , Cai L , et al. MiR‐34a derived from mesenchymal stem cells stimulates senescence in glioma cells by inducing DNA damage. Mol Med Rep. 2019;19(3):1849‐1857. doi:10.3892/mmr.2018.9800 30592284

[26] Yang P , Li QJ , Feng Y , et al. TGF‐β ‐Mir‐34a‐CCL22 signaling‐induced Treg cell recruitment promotes venous metastases of HBV‐positive hepatocellular carcinoma. Cancer Cell. 2012;22(3):291‐303. doi:10.1016/j.ccr.2012.07.023 22975373 PMC3443566

[27] Engkvist ME , Stratford EW , Lorenz S , Meza‐Zepeda LA , Myklebost O , Munthe E . Analysis of the MiR‐34 family functions in breast cancer reveals annotation error of MiR‐34b. Sci Rep. 2017;7(1):9655. doi:10.1038/s41598-017-10189-1 28848235 PMC5573726

[28] Chang TC , Wentzel EA , Kent OA , et al. Transactivation of MiR‐34a by P53 broadly influences gene expression and promotes apoptosis. Mol Cell. 2007;26(5):745‐752. doi:10.1016/j.molcel.2007.05.010 17540599 PMC1939978

[29] Qamar S , Khokhar MA , Farooq S , et al. Association of P53 overexpression with hormone receptor status and triple negative breast carcinoma. J Coll Physicians Surg Pak. 2019;29(2):164‐167. doi:10.29271/jcpsp.2019.02.164 30700357

[30] Comprehensive molecular portraits of human breast tumours. Nature. 2012;490(7418):61‐70. doi:10.1038/nature11412 23000897 PMC3465532

[31] Hsu HL , Choy CO , Kasiappan R , et al. MCT‐1 oncogene downregulates P53 and destabilizes genome structure in the response to DNA double‐Strand damage. DNA Repair. 2007;6(9):1319‐1332. doi:10.1016/j.dnarep.2007.02.028 17416211

[32] Chen C , Bai L , Cao F , et al. Targeting LIN28B reprograms tumor glucose metabolism and acidic microenvironment to suppress cancer stemness and metastasis. Oncogene. 2019;38(23):4527‐4539. doi:10.1038/s41388-019-0735-4 30742065

[33] Xiao X , Huang X , Ye F , et al. The miR‐34a‐LDHA axis regulates glucose metabolism and tumor growth in breast cancer. Sci Rep. 2016;6:21735. doi:10.1038/srep21735 26902416 PMC4763192

[34] Naeli P , Pourhanifeh MH , Karimzadeh MR , et al. Circular RNAs and gastrointestinal cancers: epigenetic regulators with a prognostic and therapeutic role. Crit Rev Oncol Hematol. 2020;145:102854. doi:10.1016/j.critrevonc.2019.102854 31877535 PMC6982584

[35] Brawner KM , Morrow CD , Smith PD . Gastric microbiome and gastric cancer. Cancer J. 2014;20(3):211‐216. doi:10.1097/ppo.0000000000000043 24855010 PMC4149312

[36] Faghihloo E , Araei Y , Mohammadi M , Mirzaei H , Mohammadi HR , Mokhtari‐Azad T . The effect of oxamflatin on the E‐cadherin expression in gastric cancer cell line. Cancer Gene Ther. 2016;23(11):396‐399. doi:10.1038/cgt.2016.52 27767089

[37] Yuan HL , Wang T , Zhang KH . Micrornas as potential biomarkers for diagnosis, therapy and prognosis of gastric cancer. Onco Targets Ther. 2018;11:3891‐3900. doi:10.2147/ott.S156921 30013369 PMC6039071

[38] Mirzaei H , Khataminfar S , Mohammadparast S , et al. Circulating micrornas as potential diagnostic biomarkers and therapeutic targets in gastric cancer: current status and future perspectives. Curr Med Chem. 2016;23(36):4135‐4150. doi:10.2174/0929867323666160818093854 27538692

[39] Simonian M , Mosallayi M , Mirzaei H . Circulating Mir‐21 as novel biomarker in gastric cancer: diagnostic and prognostic biomarker. J Cancer Res Ther. 2018;14(2):475. doi:10.4103/0973-1482.175428 29516946

[40] Jamali L , Tofigh R , Tutunchi S , et al. Circulating micrornas as diagnostic and therapeutic biomarkers in gastric and esophageal cancers. J Cell Physiol. 2018;233(11):8538‐8550. doi:10.1002/jcp.26850 29923196

[41] Peng Y , Guo JJ , Liu YM , Wu XL . MicroRNA‐34a inhibits the growth, invasion and metastasis of gastric cancer by targeting PDGFR and met expression. Biosci Rep. 2014;34(3):e00112. doi:10.1042/bsr20140020 24837198 PMC4069683

[42] Dong P , Xiong Y , Yue J , Hanley SJB , Watari H . Tumor‐intrinsic PD‐L1 signaling in cancer initiation, development and treatment: beyond immune evasion. Front Oncol. 2018;8:386. doi:10.3389/fonc.2018.00386 30283733 PMC6156376

[43] Glantz S , Gonzalez M . Effective tobacco control is key to rapid Progress in reduction of non‐communicable diseases. Lancet. 2012;379(9822):1269‐1271. doi:10.1016/s0140-6736(11)60615-6 21963004 PMC3260384

[44] Sherman EJ , Mitchell DC , Garner AL . The RNA‐binding protein SART3 promotes MiR‐34a biogenesis and G(1) cell cycle arrest in lung cancer cells. J Biol Chem. 2019;294(46):17188‐17196. doi:10.1074/jbc.AC119.010419 31619517 PMC6873168

[45] Li YL , Liu XM , Zhang CY , et al. MicroRNA‐34a/EGFR Axis plays pivotal roles in lung tumorigenesis. Oncogenesis. 2017;6(8):e372. doi:10.1038/oncsis.2017.50 28825720 PMC5608916

[46] Xue W , Dahlman JE , Tammela T , et al. Small RNA combination therapy for lung cancer. Proc Natl Acad Sci U S A. 2014;111(34):E3553‐E3561. doi:10.1073/pnas.1412686111 25114235 PMC4151750

[47] Jiang ZQ , Li MH , Qin YM , Jiang HY , Zhang X , Wu MH . Luteolin inhibits tumorigenesis and induces apoptosis of non‐small cell lung cancer cells via regulation of microRNA‐34a‐5p. Int J Mol Sci. 2018;19(2):447. doi:10.3390/ijms19020447 29393891 PMC5855669

[48] Torre LA , Bray F , Siegel RL , Ferlay J , Lortet‐Tieulent J , Jemal A . Global cancer statistics, 2012. CA Cancer J Clin. 2015;65(2):87‐108. doi:10.3322/caac.21262 25651787

[49] Sui Q , Liang J , Hu Z , et al. Genetic and microenvironmental differences in non‐smoking lung adenocarcinoma patients compared with smoking patients. Transl Lung Cancer Res. 2020;9(4):1407‐1421. doi:10.21037/tlcr-20-276 32953513 PMC7481643

[50] Louis DN , Perry A , Reifenberger G , et al. The 2016 World Health Organization classification of tumors of the central nervous system: a summary. Acta Neuropathol. 2016;131(6):803‐820. doi:10.1007/s00401-016-1545-1 27157931

[51] Osuka S , Van Meir EG . Overcoming therapeutic resistance in glioblastoma: the way forward. J Clin Invest. 2017;127(2):415‐426. doi:10.1172/jci89587 28145904 PMC5272196

[52] Hingtgen S , Figueiredo JL , Farrar C , et al. Real‐time multi‐modality imaging of glioblastoma tumor resection and recurrence. J Neurooncol. 2013;111(2):153‐161. doi:10.1007/s11060-012-1008-z 23242736 PMC3548430

[53] Sharif S , Ghahremani MH , Soleimani M . Delivery of exogenous miR‐124 to glioblastoma multiform cells by Wharton's jelly mesenchymal stem cells decreases cell proliferation and migration, and confers chemosensitivity. Stem Cell Rev Rep. 2018;14(2):236‐246. doi:10.1007/s12015-017-9788-3 29185191

[54] Huang T , Kim CK , Alvarez AA , et al. MST4 phosphorylation of ATG4B regulates autophagic activity, tumorigenicity, and radioresistance in glioblastoma. Cancer Cell. 2017;32(6):840‐55.e8. doi:10.1016/j.ccell.2017.11.005 29232556 PMC5734934

[55] Rathod SS , Rani SB , Khan M , Muzumdar D , Shiras A . Tumor suppressive miRNA‐34a suppresses cell proliferation and tumor growth of glioma stem cells by targeting Akt and Wnt signaling pathways. FEBS Open Bio. 2014;4:485‐495. doi:10.1016/j.fob.2014.05.002 PMC4060015 24944883

[56] Li WB , Ma MW , Dong LJ , Wang F , Chen LX , Li XR . MicroRNA‐34a targets notch1 and inhibits cell proliferation in glioblastoma Multiforme. Cancer Biol Ther. 2011;12(6):477‐483. doi:10.4161/cbt.12.6.16300 21743299

[57] Yin D , Ogawa S , Kawamata N , et al. Mir‐34a functions as a tumor suppressor modulating EGFR in glioblastoma Multiforme. Oncogene. 2013;32(9):1155‐1163. doi:10.1038/onc.2012.132 22580610 PMC4085050

[58] Wang X , Wang HK , McCoy JP , et al. Oncogenic HPV infection interrupts the expression of tumor‐suppressive miR‐34a through viral oncoprotein E6. RNA. 2009;15(4):637‐647. doi:10.1261/rna.1442309 19258450 PMC2661824

[59] Wang P , Zhai G , Bai Y . Values of miR‐34a and miR‐218 expression in the diagnosis of cervical cancer and the prediction of prognosis. Oncol Lett. 2018;15(3):3580‐3585. doi:10.3892/ol.2018.7791 29456728 PMC5795828

[60] Zhang R , Su J , Xue SL , et al. HPV E6/P53 mediated Down‐regulation of miR‐34a inhibits warburg effect through targeting LDHA in cervical cancer. Am J Cancer Res. 2016;6(2):312‐320. 27186405 PMC4859662

[61] Enwere EK , Kornaga EN , Dean M , et al. Expression of PD‐L1 and presence of CD8‐positive T cells in pre‐treatment specimens of locally advanced cervical cancer. Mod Pathol. 2017;30(4):577‐586. doi:10.1038/modpathol.2016.221 28059093

[62] Meng Y , Liang H , Hu J , et al. PD‐L1 expression correlates with tumor infiltrating lymphocytes and response to Neoadjuvant chemotherapy in cervical cancer. J Cancer. 2018;9(16):2938‐2945. doi:10.7150/jca.22532 30123362 PMC6096364

[63] Liu Y , Jiang J , Liu C , et al. Synergistic anti‐tumor effect of anti‐PD‐L1 antibody cationic microbubbles for delivery of the miR‐34a gene combined with ultrasound on cervical carcinoma. Am J Transl Res. 2021;13(3):988‐1005. 33841635 PMC8014418

[64] Córdova‐Rivas S , Fraire‐Soto I , Mercado‐Casas Torres A , et al. 5p and 3p strands of miR‐34 family members have differential effects in cell proliferation, migration, and invasion in cervical cancer cells. Int J Mol Sci. 2019;20(3):545. doi:10.3390/ijms20030545 30696040 PMC6387060

[65] Momen‐Heravi F , Bala S . Extracellular vesicles in oral squamous carcinoma carry oncogenic miRNA profile and reprogram monocytes via NF‐ΚB pathway. Oncotarget. 2018;9(78):34838‐34854. doi:10.18632/oncotarget.26208 30410681 PMC6205181

[66] Yoon AJ , Wang S , Kutler DI , et al. MicroRNA‐based risk scoring system to identify early‐stage oral squamous cell carcinoma patients at high‐risk for cancer‐specific mortality. Head Neck. 2020;42(8):1699‐1712. Epub 2020/01/26. doi:10.1002/hed.26089 31981257 PMC7369212

[67] Wang Y , Chen J , Chen X , et al. Mir‐34a suppresses HNSCC growth through modulating cell cycle arrest and senescence. Neoplasma. 2017;64(4):543‐553. doi:10.4149/neo_2017_408 28485160

